# Cathodal Transcranial Direct Current Stimulation Does Not Change Implicit Associations Against Alcohol in Alcohol Use Disorder: A Preregistered Clinical Trial

**DOI:** 10.1111/adb.70029

**Published:** 2025-03-12

**Authors:** Tobias Schwippel, Philipp A. Schroeder, Janik Philipp, Simone Weller, Christian Plewnia

**Affiliations:** ^1^ Department of Psychiatry and Psychotherapy, Neurophysiology and Interventional Neuropsychiatry University of Tübingen Tübingen Germany; ^2^ Department of Psychiatry University of North Carolina at Chapel Hill Chapel Hill North Carolina USA; ^3^ Carolina Center for Neurostimulation University of North Carolina at Chapel Hill Chapel Hill North Carolina USA; ^4^ Department of Psychology University of Tübingen Tübingen Germany; ^5^ German Center for Mental Health (DZPG), Partner Site Tübingen Germany

**Keywords:** alcohol use disorder, cathodal tDCS, craving, dual process model, implicit association test, transcranial direct current stimulation

## Abstract

Addictive behaviour is shaped by the dynamic interaction of implicit, bottom‐up and explicit, top‐down cognitive processes. In alcohol use disorder (AUD), implicit alcohol‐related associations have been shown to predict increased subsequent alcohol consumption and are linked to the risk of relapse. Explicit cognitive processes, exerting prefrontal top‐down control, are particularly significant during the critical period following the decision to abstain. This study aims to map implicit and explicit cognitive processes in recently abstinent individuals with AUD and to explore the effect of cathodal transcranial direct current stimulation (tDCS) on implicit associations by modulating top‐down control. In this preregistered, double‐blind, sham‐controlled clinical trial, 30 abstinent individuals with AUD participated in two experimental sessions. They received either 2 mA cathodal tDCS to the left dorsolateral prefrontal cortex (dlPFC) or sham tDCS in a crossover design. During tDCS, participants completed the alcohol approach implicit association test (IAT) and the drinking identity IAT, along with two control tasks. Additionally, we collected explicit ratings of the IAT stimuli and assessed craving before and after each experimental session. Preregistered ANOVAs revealed significant implicit alcohol–avoidance and non–drinking identity biases. Cathodal tDCS did not modulate IAT scores. Explicit ratings showed a preference for non‐alcoholic drinks and non‐drinking identity, correlating moderately with IAT scores. Exploratory analyses indicated that cathodal tDCS mitigated the increase in nicotine craving during the experimental session. This preregistered clinical trial provides robust evidence that single‐session cathodal tDCS to the left dorsolateral prefrontal cortex does not modulate implicit associations in AUD, with Bayesian analyses corroborating the absence of tDCS effects. Our results emphasize the impact of contextual factors on the interplay between explicit and implicit cognitive processes and underscore the importance of investigating multisession stimulation paradigms in future research.

## Graphical abstract

This preregistered, double‐blind, sham‐controlled study investigated the effect of cathodal tDCS over the left dlPFC on implicit alcohol‐related associations in abstinent individuals with AUD. While participants showed implicit alcohol‐avoidance and non‐drinking identity biases, tDCS did not modulate these effects. Exploratory analyses revealed that cathodal tDCS mitigated the increase in nicotine craving during the experimental session.

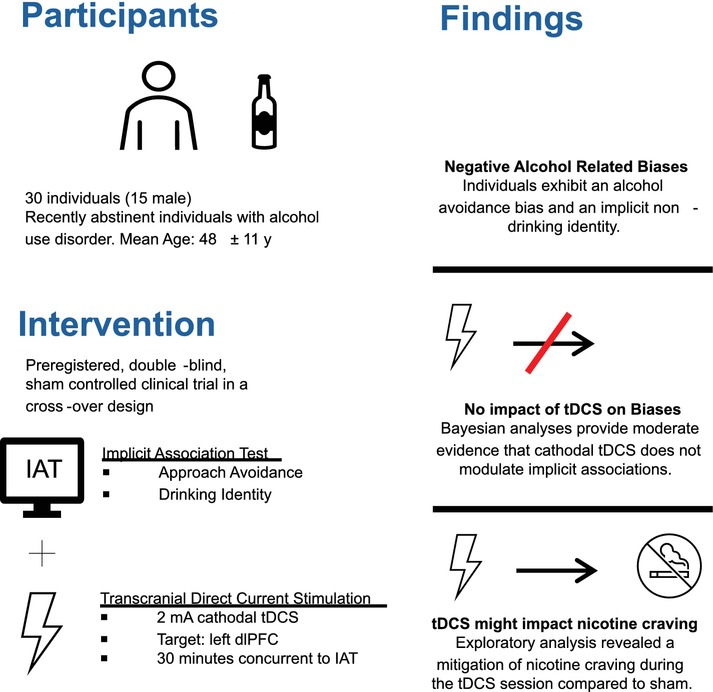

## 1 Introduction

Cognitive processes play a pivotal role in aetiology, maintenance and treatment of drug addiction [1]. The dual process model posits two distinct cognitive processes: a fast, impulsive, bottom‐up process, and a slow, reflective, controlled, top‐down process [2]. For alcohol use disorder (AUD), this model highlights the dynamic interaction between a dominant bottom‐up system and a maladaptive top‐down control system, leading to drug‐seeking behaviour, craving and compulsion [3, 4, 5]. Despite the established role of cognitive processes in AUD, there is a significant gap in understanding these processes at specific stages of the addiction cycle. Additionally, it remains unclear how non‐invasive brain stimulation (NIBS) can be utilized to effectively modulate the dynamic interplay between the bottom‐up and top‐down systems in AUD.

Reward‐related activation of the dopaminergic midbrain system, comprising the striatum, amygdala, nucleus accumbens and hippocampus, in response to drug cues is hypothesized to reflect dominant bottom‐up processing, leading to addictive behaviour [6, 7]. A cognitive task to assess bottom‐up processing is the implicit association test (IAT) [8]. It is thought to measures bottom‐up, implicit cognition through response times in a double‐classification task, with scores reflecting the strength of associations between target and attribute categories [9, 10]. However, the IAT's psychometric properties are debated [11, 12], and it increasingly is recognized that the test also captures explicit cognitive processes including situational factors [13]. In the context of alcohol consumption, different versions of the IAT have been largely used in a non‐clinical population. It has been shown that implicit measures correlate with drinking outcomes over time, suggesting their potential as vulnerability markers for problem drinking [14]. Likewise, the recently developed drinking identity IAT, which measures self‐related cognition in AUD with reduced social desirability bias [15, 16], has demonstrated stability over time in predicting risk drinking behaviour among college students [17, 18]. For individuals with AUD, the presence of negative implicit attitudes towards alcohol as compared with soft drinks has been reported [19]. This is consistent with our previous IAT findings in recently abstinent individuals with AUD, which revealed negative implicit associations towards alcohol in the drinking identity IAT and the alcohol approach IAT [20], emphasizing the critical role of situational factors on implicit processes. Nevertheless, the current body of research is limited by a lack of studies conducted in clinical populations of AUD, and the reproducibility of findings remains a significant challenge.

Besides alteration in the mesolimbic bottom‐up system, dysfunction of the prefrontal cortex (PFC) is hypothesized to influence addiction through changes in cognitive and emotional processes [21, 22]. In particular, impairments in response inhibition and salience attribution have been closely linked to addictive behaviours [23]. This has been demonstrated in neuroimaging studies, showing increased engagement of prefrontal executive functions and top‐down control during drug cue exposure, and blunted response to non‐drug‐related cues [24]. Importantly, the midbrain bottom‐up and prefrontal top‐down processes closely interact via meso–cortico–striatal pathways, and the balance between both processes dynamically changes during the addiction cycle [25]. Given this interplay, NIBS techniques hold the promise of directly disrupting maladaptive prefrontal activity, thereby modulating the PFC's influence on bottom‐up processes, as measured by the IAT.

Transcranial direct current stimulation (tDCS) can be administered using either inhibitory cathodal or excitatory anodal stimulation [26]. When targeting PFC activity in AUD, both inhibitory paradigms to disrupt maladaptive prefrontal function in response to drug cues and excitatory protocols to strengthen top‐down processes in non–drug‐related situations are viable approaches. For example, the enhancement of top‐down control is often the rationale for employing tDCS to reduce craving in AUD. However, results have been mixed; a recent meta‐analysis did not find significant effects of tDCS on alcohol craving [27], whereas reductions in craving for tobacco and other drugs were observed in a recent analysis [28]. A similar rationale to increase top‐down control is employed in most studies combining the IAT with concurrent anodal tDCS, aiming to reduce the IAT effect. Successful modulation of implicit associations has been reported in several contexts, including racial prejudices [29], gender bias [30, 31] and attitudes towards food in eating disorders [32]. Yet, in the context of AUD, Gladwin and colleagues were unable to confirm a significant effect of anodal tDCS on the IAT effect [33]. A follow‐up study in student drinkers revealed that while prefrontal anodal tDCS did reduce craving, it did not influence IAT scores in two separate IATs [34]. Furthermore, a clinical trial with 98 individuals with AUD revealed that four sessions of combined attentional bias modification and bifrontal tDCS did not result in significant effects on implicit associations [35]. Research using cathodal tDCS [36] for reducing prefrontal activity and its effects on the IAT is comparatively limited. The general viability of this approach was shown by Schroeder and colleagues, who reported and reduction of the IAT effect through cathodal tDCS in the flower‐insect IAT [37]. However, the preregistered first clinical trial in recently abstinent individuals with AUD did not find an effects of 1 mA cathodal tDCS on IAT scores in two alcohol‐related IATs and the flower‐insect IAT [20]. It was discussed that achieving behavioural effects in patient populations might necessitate higher current intensities, particularly due to the impact of medication and structural brain changes in individuals with AUD, as is known from other neuropsychiatric disorders [38, 39]. Therefore, further research is needed to determine if cathodal tDCS to the PFC can effectively reduce the IAT effect in individuals with AUD.

To address this gap, our preregistered clinical trial employed a higher current intensity of cathodal tDCS to modulate prefrontal activity and implicit cognitive processes in individuals with AUD. We hypothesized that cathodal tDCS would reduce an existing alcohol bias in the IAT. Additionally, we aimed to replicate the implicit alcohol avoidance bias and non‐drinking identity observed in our previous study, ensuring the robustness and reliability of our findings. We also analysed explicit associations and their relationship to IAT task performance, as well as the potential impact of tDCS on nicotine and alcohol craving. Ultimately, the primary goal of this study was to assess whether cathodal tDCS is effective in modulating these cognitive biases, providing clarity to the field and allowing for more informed future research directions.

## 2 Methods

### 2.1 Experimental Design

This is a preregistered, double‐blinded, randomized, controlled study using a crossover design (https://osf.io/wd6em). We included individuals diagnosed with AUD who were currently abstinent. These participants were recruited from the Addiction Clinic Tübingen. The study was approved by the local ethics committee (877/2017BO2). Before inclusion, all participants signed informed consent and received monetary compensation.

**FIGURE 1:**
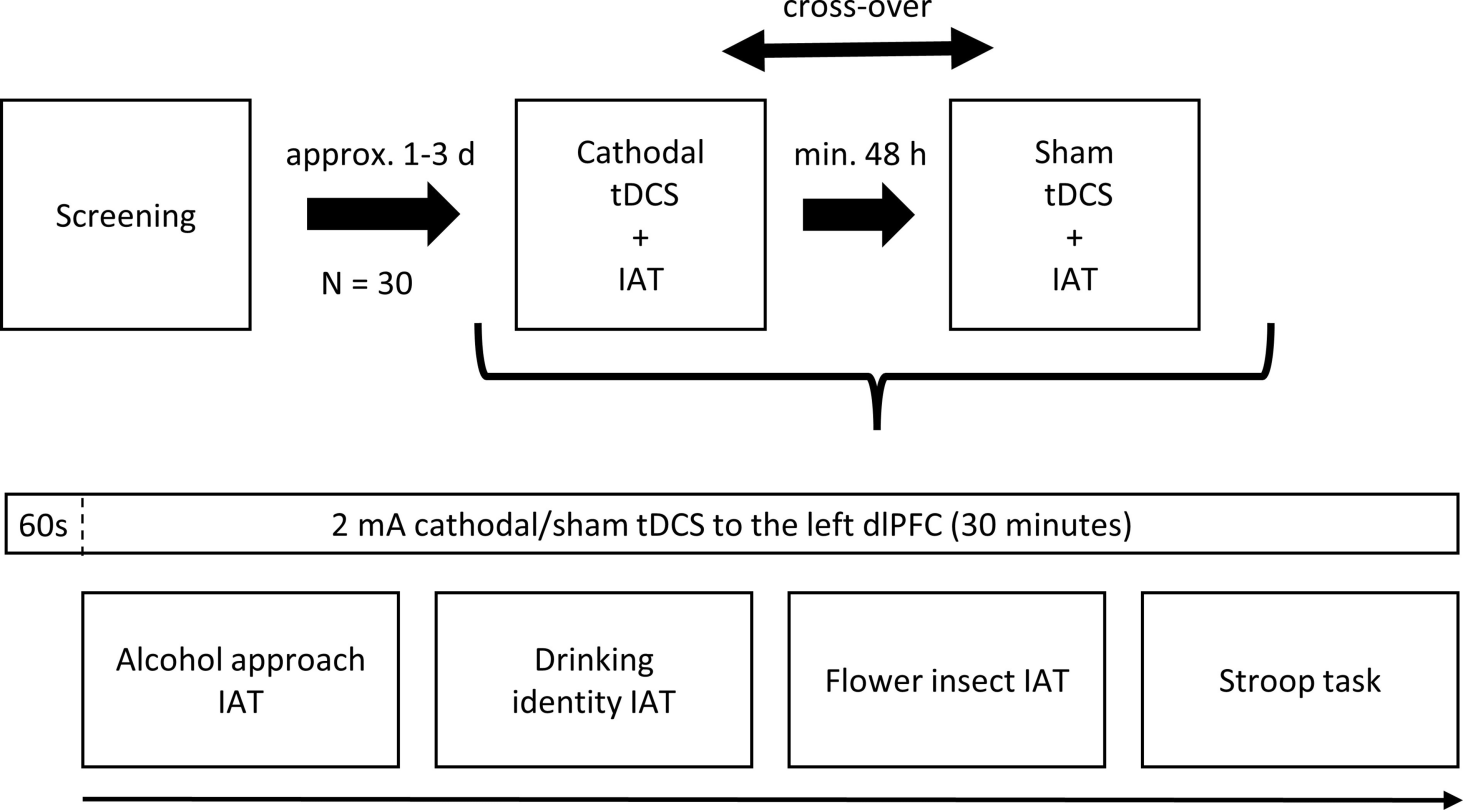
Study design. Randomized sham‐controlled clinical trial in a cross‐over design.

The experiment consisted of three sessions, with the first session being a screening session to collect demographic and clinical data. During the subsequent two experimental sessions, participants underwent two randomized stimulation conditions (cathodal and sham), with at least 48 h between sessions. Throughout the experimental sessions, participants completed two alcohol‐related IATs (alcohol approach IAT and drinking identity IAT), a flower‐insect IAT and the Stroop task, leading to a total session duration of approximately 30 min. The task sequence was not randomized (Figure 1).

### 2.2 Study Population

The required sample size was determined through a preregistered power analysis using MorePower 6.0.3 software. The analysis focused on the critical 2 × 2 interaction term (IAT block type × stimulation condition) in the ANOVA. Based on the effect size (partial η^2^ = 0.25) reported in the most closely related publication [37], we aimed to achieve a statistical power of 80% (1 − β = 0.80) with a significance level of α = 0.05. The power analysis indicated a minimum sample size of 27 participants. To account for an anticipated 10% dropout rate, we recruited 30 participants (15 female) from the Addiction Clinic Tübingen. Inclusion criteria were age 18–65 years, capacity to give informed consent, clinically diagnosed alcohol dependence according to ICD‐10 (F10.2), current abstinence, proficient knowledge of the German language, right‐handedness. Exclusion criteria were history of bipolar disorder, schizophrenia, seizures, severe head injury or traumatic brain injury, metallic objects in the head (e.g., cochlear implant and aneurysm clip), pacemaker, pregnancy according to self‐report, use of antiepileptic or benzodiazepine medication > 1 mg lorazepam equivalent, suicidality. Table 1 summarizes demographic and clinical characteristics.

**TABLE 1:** Demographic and clinical data.

	*N*	M	SD	Min	Max
Gender					
Female	15				
Male	15				
Age (year)		48	11	24	64
School education (year)		10.8	1.7	8	14
Education total (year)		15.4	3.3	9	21.5
Abstinence (day)		17.3	12.1	4	49
Inpatient Treatment		3.5	4.1	0	20
Longterm therapy		0.9	1	0	4
AUDIT		27.5	7.0	10	40
Craving (NAS 1–10)		6.7	1.9	2	10
Smoking status/FTND					
Smoker	21	3.2	3.2	0	10
Non‐smoker	9				
MWT‐B (IQ)		101	13	86	130
BIS‐11		67.7	6.9	58	88
BDI		15.8	10.3	0	43

Abbreviations: AUDIT: Alcohol Use Disorder Identification Test; BDI: Beck Depression Inventory; BIS‐11: Barratt Impulsiveness Scale; M: mean; MWT‐B: Mehrfach Wortschatz Intelligenztest (premorbid intelligence); NAS: Numeric Analog Scale, FTND: Fagerstrom Test for Nicotine Dependence; M: mean; SD: standard deviation.

### 2.3 Transcranial Direct Current Stimulation

We utilized the neurConn DC‐Stimulator Plus (neuroConn GmbH, Ilmenau) for our study. The 35 cm^2^ tDCS electrode (rectangular, 7 × 5 cm) was placed on the left dlPFC, in accordance with the 10–20 EEG system (F3), and the anode was attached to the right deltoid muscle. Active cathodal tDCS involved stimulating the targeted areas with 2 mA for 30 min, including a 15 s fade‐in and fade‐out period. In contrast, sham stimulation used the manufacturer's sham mode, delivering 2 mA for 90 s including a 15 s ramp up/down. The IAT began 60 s after the start of active/sham tDCS stimulation, ensuring minimal overlap between the sham stimulation and the task. After completion of the three IATs and the Stroop task, participants were instructed to remain seated until the duration of the stimulation was fully elapsed. Throughout the course of the experiment, the impedance was consistently maintained below 10 kΩ.

### 2.4 Implicit Association Test

The experimental sessions included three computer‐based IATs administered to each participant in a fixed sequence. Tasks were programmed using PsychoPy 1.81.02.35 [40]. The detailed task description can be retrieved from our previous publication [20]. In short, the words representing attribute stimuli (e.g., ‘drinking’) were displayed in light‐green font, while target stimuli (e.g., ‘me’) were shown in white font to enhance distinction. The IATs comprised seven blocks, progressively introducing task guidelines and conditions. The tasks contained congruent test blocks and incongruent blocks. Participants were instructed to respond quickly and accurately to stimuli presented at the centre of the screen by pressing corresponding keyboard keys (Figure S1). Incorrect responses required a manual correction. All stimuli used are available in the supplementary material.

### 2.5 Stroop Task

The Stroop task was administered after completion of all IATs [41]. Participants were instructed to respond to a word appearing in the centre of the screen by pressing the keyboard key in the corresponding colour. The displayed word and colour matched in 50% of the trials (congruent), while in the other 50%, the colour and word were incongruent. We report the ‘Stroop’ factor (incongruent vs. congruent) as well as the stimulation condition (sham vs. tDCS).

### 2.6 Explicit Alcohol‐Related Cognition

After each experimental session, explicit measures of alcohol‐related cognition were assessed by completing a 7‐item Likert scale. Participants could choose between *very repelled* (1) and *very attractive* (7) regarding approach/avoidance tendencies for each soft drink and alcoholic beverage previously presented in the alcohol approach IAT. Explicit assessments regarding drinking identity were collected in the same manner, with participants selecting between the ratings *not me at all* (1) and *completely me* (7) for all stimuli presented in the IAT. We analysed data from the sham session to avoid potential tDCS effects. The difference score between negative and positive explicit ratings (e.g., alcoholic beverages − soft drinks) was calculated for each participant.

### 2.7 Questionnaires

During the screening session, we collected demographic data and administered the Alcohol Use Disorders Identification Test (AUDIT) [42], the Barratt Impulsiveness Scale (BIS‐11) [43] and Beck's Depression Inventory (BDI) [44]. Additionally, current craving was assessed using a Numeric Analog Scale (NAS) before and after each experimental session, and adverse stimulation effects were collected with a 7‐item Likert scale.

### 2.8 Statistical Analysis

The analyses adhered to the preregistered methodology and utilized the same statistical model for the alcohol‐related IATs and the control tasks. Response times (RTs) above ± 2 standard deviations were excluded as outliers. Data were excluded if more than 10% of all responses were faster than 300 ms. The average correct RTs and error rates were used as dependent variables in separate ANOVAs, encompassing the repeated factors IAT (congruent vs. incongruent block) × Trial Type (target vs. attribute trials) × Stimulation (cathodal vs. sham tDCS). Consistent with preregistration, the interaction between IAT and stimulation was our primary outcome, as this would indicate a modulation of implicit associations by tDCS. Explicit ratings were analysed with paired *t*‐tests. Furthermore, we present Bayes factors (BF_01_) to gauge the absence of corroborative evidence for the effects of stimulation [45]. For further examination of the relationship between neuropsychological factors and alcohol related IAT, an analysis of several covariates of alcohol pathology (AUDIT score, duration of abstinence) was conducted. To perform correlation analysis, D‐IAT scores were computed. Correction for multiple comparisons was employed for our preregistered hypotheses, whereas exploratory analyses remained uncorrected.

## 3 Results

### 3.1 IAT Effect

Significant IAT effects were detected for the alcohol approach IAT (*F* (1, 28) = 15.16, *p* = 0.001, *η*_*p*_^2^ = 0.35) as well as for the drinking identity IAT (*F*(1, 29) = 8.11, *p* = 0.008, *η*_*p*_^2^ = 0.22). Precisely, faster response times in the IAT‐incompatible block (alcohol + avoidance; nondrinker + me) compared with the IAT‐compatible block (alcohol + approach; drinker + me) were observed (Table 2). This indicates an implicit alcohol‐avoidance bias and an implicit non‐drinker identity. As hypothesized, significant faster reaction times were observed in the IAT compatible block (flower + positive; insect + negative) compared with the IAT incompatible block (flower + negative; insect + positive) in the flower‐insect IAT (*F*(1, 29) = 66.26, *p* < 0.001*, η*_*p*_^2^ = 0.70).

**TABLE 2:** Response times and D‐IAT scores of IAT tasks.

Task	tDCS	Response time (ms) by IAT condition				D‐IAT score	
		Congruent		Incongruent		M	SD
		M	SD	M	SD		
Alcohol approach IAT	Sham	1261	486	1080	330	−0.349	0.419
	Cathodal	1302	481	1122	393	−0.287	0.482
Drinking identity IAT	Sham	1168	407	962	169	−0.206	0.490
	Cathodal	1204	345	981	197	−0.208	0.416
Flower insect IAT	Sham	815	179	972	261	0.582	0.284
	Cathodal	807	147	951	229	0.507	0.243
Stroop task	Sham	766	117	830	118	—	—
	Cathodal	749	121	815	129	—	—

*Note:* M and SD indicate the mean and standard deviation. D‐IAT scores were computed using the scoring algorithm by Greenwald et al. [9]. A negative D‐IAT score implies a stronger response for incongruent trials. It should be noted that this algorithm is not applicable to the Stroop task.

### 3.2 Cathodal tDCS Effects

There was no significant two‐way interaction between stimulation condition and IAT effect in all three IAT tasks, *F* < 0.96, *p >* 0.337, *η*_*p*_^2^ < 0.04. Bayesian analyses yielded moderate evidence in favour of the null hypothesis (i.e., omission of the tDCS × IAT interaction; 4.14 < BF_01_ < 5.38). Also, there was no significant three‐way interaction of IAT effects and condition with AUDIT scores, duration of abstinence, or smoking dependence, all *F*s < 2.3 (detailed results in the supplementary material).

### 3.3 Clinical Ratings and D‐IAT Scores

Neither severity of addiction (AUDIT), days of abstinence, impulsiveness (BIS‐11) nor depression (BDI) was significantly correlated with D‐IAT scores (Table 3).

**TABLE 3:** Correlations of D‐IAT scores with psychopathology, demography and explicit ratings.

Variable	M	SD	1	2	3	4	5	6	7	8	9	10
1. Alcohol approach IAT	−0.35	0.42										
2. Drinking identity IAT	−0.21	0.49	0.18									
			[−0.20, 0.51]									
3. Flower insect IAT	0.58	0.28	0.12	−0.11								
			[−0.25, 0.47]	[−0.45, 0.26]								
4. Abstinence [d]	17.37	12.32	−0.15	0.01	**−0.46** *							
			[−0.49, 0.23]	[−0.35, 0.37]	**[−0.70, ‐0.12]**							
5. AUDIT	27.53	7.15	0.15	0.12	−0.07	0.04						
			[−0.23, 0.49]	[−0.25, 0.46]	[−0.42, 0.30]	[−0.32, 0.40]						
6. FTND	4.62	2.91	0.02	0.12	−0.18	0.11	**0.51** *					
			[−0.41, 0.45]	[−0.33, 0.53]	[−0.57, 0.27]	[−0.34, 0.52]	**[0.11, 0.77]**					
7. Age [years]	48.37	11.43	0.20	**−0.41** *	−0.04	−0.19	−0.29	−0.29				
			[−0.18, 0.53]	**[−0.67, ‐0.06]**	[−0.40, 0.32]	[−0.52, 0.18]	[−0.59, 0.08]	[−0.64, 0.16]				
8. BIS‐11	67.60	7.11	−0.02	0.11	0.02	0.12	0.29	−0.01	−0.23			
			[−0.39, 0.35]	[−0.26, 0.45]	[−0.34, 0.38]	[−0.25, 0.46]	[−0.08, 0.59]	[−0.44, 0.42]	[−0.54, 0.14]			
9. BDI	15.86	10.52	−0.09	−0.24	−0.12	0.10	**0.50** **	**0.51** *	−0.20	0.01		
			[−0.45, 0.30]	[−0.56, 0.14]	[−0.47, 0.26]	[−0.29, 0.45]	**[0.15, 0.74]**	**[0.08, 0.78]**	[−0.53, 0.19]	[−0.37, 0.38]		
10. Explicit beverage rating	−2.85	1.83	0.20	**0.41** *	−0.11	−0.05	−0.09	−0.11	−0.07	−0.09	−0.24	
			[−0.18, 0.53]	**[0.06, 0.67]**	[−0.45, 0.26]	[−0.40, 0.32]	[−0.43, 0.28]	[−0.51, 0.34]	[−0.42, 0.30]	[−0.44, 0.28]	[−0.56, 0.15]	
11. Explicit drinking identity	−3.41	2.24	0.35^a^	0.13	0.10	−0.01	0.12	−0.05	−0.12	−0.03	0.11	**0.52** **
			[−0.02, 0.64]	[−0.24, 0.47]	[−0.27, 0.44]	[−0.37, 0.35]	[−0.26, 0.46]	[−0.47, 0.39]	[−0.46, 0.25]	[−0.38, 0.34]	[−0.28, 0.46]	**[0.20, 0.74]**

*Note:* Pearson's correlation coefficient. Values in square brackets indicate the 95% confidence interval for each correlation.

Abbreviations: AUDIT: Alcohol Use Disorder Identification Test; BDI: Beck Depression Inventory; BIS‐11: Barratt Impulsiveness Scale; FTND: Fagerstrom Test for Nicotine Dependence; M: mean; SD: standard deviation.

^a^ indicates *p* < 0.07.

* indicates *p* < 0.05.

** indicates *p* < 0.01.

### 3.4 Stroop Task

A repeated measures ANOVA with the factors Stroop and stimulation condition returned a significant main effect of Stroop (*F*(1, 29) = 98.93, *p* < 0.001, *η*_*p*_^2^ = 0.77). The two‐way interaction between Stroop and stimulation condition was not significant (*F*(1, 29) = 2.81, *p* = 0.105, *η*_*p*_^2^ = 0.09). As hypothesized, there was no significant main effect of tDCS (*F*(1, 29) = 0.31, *p* = 0.585, *η*_*p*_^2^ = 0.01).

### 3.5 Explicit Alcohol‐Related Cognition

For the alcohol approach IAT, pictures of soft drinks (M = 5.20, SD = 1.04) were rated more positively than pictures of alcoholic beverages (M = 2.35, SD = 1.55). Similarly, in the drinking identity IAT, non‐drinking identity descriptions (M = 5.65, SD = 1.20) were rated higher than drinking identity words (M = 2.24, SD = 1.41) Thus, the direction of explicit rating mirrors results from the IAT. Interestingly, explicit ratings of alcoholic beverages were significantly correlated with D‐IAT scores from the drinking identity IAT (*r* = 0.41, *p* = 0.024), whereas explicit drinking identity ratings were positively correlated with D‐IAT scores of the alcohol approach IAT (*r* = 0.35, *p* = 0.062). This replicates correlation results from our previous study. For all correlation results, consult Table 3.

### 3.6 Craving

Craving was assessed by NAS ranging from 1 (*no craving*) to 10 (*maximal craving*) directly before and after each experimental session. In both experimental conditions, alcohol craving significantly decreased at the end of the session, but no significant difference between groups was observed. In contrast, nicotine craving was only significantly elevated after sham tDCS, but not after cathodal tDCS (Table 4). Consequently, Kruskal–Wallis test on nicotine craving difference scores revealed a significant effect of condition (*χ* ^*2*^ = 3.91, *p* = 0.048), indicating that cathodal tDCS potentially inhibited an increase in nicotine craving.

**TABLE 4:** Alcohol and nicotine craving.

Substance	tDCS	Pre		Post		*Δ*	*z*	*p*
		M	SD	M	SD			
Alcohol	Sham	1.53	0.90	1.23	0.50	−0.30	2.20	0.031*
	Cathodal	1.83	1.32	1.37	0.62	−0.47	2.17	0.033*
Nicotine	Sham	2.36	1.73	3.82	2.30	1.45	−2.67	0.008*
	Cathodal	3.64	2.67	3.96	2.77	0.32	−1.12	0.269

*Note:* Δ = post session − pre session craving scores. Wilcoxon signed‐rank test.

* *p* < 0.05.

### 3.7 Tolerability of tDCS

The adverse stimulation effect questionnaire consisted of a 5‐point Likert scale (1 = *not at all* to 5 = *extremely*) with seven items (headache, fatigue, metallic taste, itching, pain, heat and burning sensations (Table 5). All adverse effects were averaged, resulting in overall mild adverse effect severity after cathodal (M = 1.39, SD = 0.78) and after sham tDCS (M = 1.45, SD = 0.91). This underlines that 2 mA cathodal tDCS is safe and that participants experienced expected, mild adverse stimulation effects irrespective of group allocation.

**TABLE 5:** Adverse stimulation effects.

Adverse effect	Cathodal		Sham	
	M	SD	M	SD
Headache	1.03	0.18	1.07	0.25
Metal taste	1.07	0.25	1.27	0.78
Burning	1.70	0.95	1.77	1.14
Itching	2.13	1.11	2.47	1.28
Tired	1.20	0.48	1.10	0.31
Pain	1.17	0.46	1.23	0.57
Heat	1.40	0.81	1.27	0.58

*Note:* Adverse stimulation effects scored by individuals on a 5‐item Likert scale (0 = *not at all*, 5 = *extremely*).

### 3.8 Blinding

After each session, participants were inquired if they received a verum stimulation as opposed to a sham stimulation which only tingled. After cathodal tDCS, 27/30 participants indicated they had received verum tDCS. Following sham stimulation, 24/30 participants thought they had received verum tDCS. Consequently, a chi‐square test of independence showed no significant effect of group allocation (*χ* ^2^(1, *N* = 30) = 1.17, *p* = 0.279). Therefore, the blinding procedure for 2 mA cathodal tDCS can be considered successful.

## 4 Discussion

In this preregistered, double‐blind, sham‐controlled clinical trial, we successfully replicated an implicit bias towards alcohol avoidance and non‐drinking identity in recently abstinent individuals with AUD. In accordance with these implicit findings, explicit ratings of task stimuli indicated a preference for non‐alcoholic drinks and a non‐drinking identity. Weak to moderate correlations were observed between explicit ratings and D‐IAT scores, highlighting the interconnectedness of explicit and implicit cognitive processes. No effect of 2 mA cathodal tDCS to the left dlPFC on IAT scores was found. However, our exploratory analysis suggested that 2 mA cathodal tDCS may have mitigated the expected increase in nicotine craving during the 60 min study intervention. These findings further confirm the presence of negative alcohol‐related biases in recently abstinent individuals with AUD and their resistance to modulation by single‐session cathodal tDCS.

### 4.1 Implicit and Explicit Associations

Our study revealed alcohol‐averse implicit associations in both IATs, replicating our previous findings with an independent sample [20] and corroborating results from other studies [46, 47]. The direction of the IAT effects can be explained by situational factors which shaped explicit cognition subsequently impacting implicit associations as measured in the IAT [48, 49, 50]. This hypothesis is in line with a dual‐process model of addiction which acknowledges the dynamic interplay of implicit and explicit cognitive processes which are both shaped by situational factors [5, 51]. This is especially relevant in our study sample, which consisted of abstinent individuals with AUD who had recently participated in therapeutic interventions. These included psychotherapy, peer‐to‐peer contact and psychoeducation, all aimed at reshaping explicit cognition in disfavour of alcohol. As a result, we observed the expected negative explicit associations towards alcohol‐related stimuli from the IAT. These explicit associations demonstrated a weak to moderate correlation with implicit associations, a correlation stronger than those reported in two meta‐analyses of IATs [52, 53], highlighting the potential impact of explicit cognition through top‐down processes on implicit associations. Thus, our findings highlight the dynamic interplay of cognitive processes at a crucial moment in an individual's addiction cycle.

### 4.2 Effect of tDCS

Informed by previous studies indicating that higher current intensities may be required to elicit behavioural effects in patient populations [38, 39], we employed a current of 2 mA. However, we did not detect a tDCS effect at this intensity. Bayesian analysis provided moderate evidence that 2 mA cathodal tDCS is ineffective in modulating implicit associations in individuals with AUD. One possible explanation is the non‐linear excitability change reported with cathodal tDCS. Increasing the stimulation intensity to 2 mA might reverse the expected inhibition, resulting in increased excitability [54, 55]. These non‐linear effects could be further complicated by structural and functional alcohol‐related brain damage [56]. Additionally, although we increased the current intensity during this single session, effective modulation of implicit associations might require multiple tDCS sessions to induce necessary secondary neuroplastic effects. A recent clinical trial (*n* = 125) in recently abstinent individuals with AUD combined five sessions of tDCS with alcohol cue inhibitory control training and reported a significantly higher abstinence rate at the 2‐week follow‐up, but no long‐term benefits [57]. Similarly, in other mental health conditions, transcranial magnetic [58] or transcranial electric stimulation protocols [59] are typically administered over several days or weeks, emphasizing the critical importance of repeated stimulation sessions to achieve measurable behavioural and clinical outcomes. Another approach to increase effectiveness could be the individualization of stimulation based on ongoing neurophysiological activity, as recently demonstrated in a study in depression [59]. Lastly, in our specific study sample, implicit associations in favour of alcohol were not present in the IAT, possibly due to situational factors. This lower margin for improvement limits the potential effectiveness of tDCS in reducing alcohol‐related implicit associations (floor effect).

### 4.3 Craving

Alcohol craving was minimal at the start of the experimental session, suggesting a floor effect that limited the potential for further reduction. Nonetheless, craving scores decreased slightly after both sham and 2 mA cathodal tDCS. In contrast, among the subset of 21 smoking participants, nicotine craving was more pronounced before the experimental sessions. Consistent with our previous study, we anticipated an increase in nicotine craving in both conditions following the experimental session [20]. However, after 2 mA cathodal tDCS, nicotine craving was not significantly increased suggesting a potential alleviating effect of cathodal tDCS on nicotine craving. While this was not a preregistered outcome, it carries potential clinical importance, as craving remains a pervasive and challenging aspect of addiction treatment. The observed reduction in craving following cathodal tDCS aligns with a recent meta‐analysis reporting a medium effect size for bifrontal tDCS with the cathode on the left dlPFC in reducing craving across various substances [28]. Interestingly, consistent with the findings of this study, no significant effect of tDCS on alcohol craving was observed. Another meta‐analysis highlighted that higher stimulation intensities (2 mA) yielded greater effect sizes compared with lower intensities (1 mA), further supporting our results [60]. Mechanistically, the reduction in craving may be attributed to the decreased cortical excitability and long‐term depression (LTD)‐like effects of cathodal tDCS, which potentially modulate the frontal‐striatal circuit [61].

### 4.4 Limitations

While our sample size was determined by a preregistered power analysis and is adequate for a clinical trial employing NIBS, and the cross‐over design enhances statistical power, we recognize that subtle tDCS effects on the IAT may have remained undetected. Additionally, the timing of the study intervention—conducted shortly after detoxification and specialized addiction treatment—may have introduced negative alcohol‐related biases, potentially resulting in a floor effect that limited further modulation by cathodal tDCS. However, the immediate period following abstinence is a critical moment in an individual's recovery of addiction, and understanding its cognitive processes is relevant. Also, while we assessed the implicit self‐concept with the drinking identity IAT and measured explicit ratings of all stimuli used in the IAT, we did not employ an explicit self‐concept measure like the Alcohol Self‐Concept Scale [17]. Lastly, we did not incorporate neurophysiological measures, which might have detected neurophysiological effects of tDCS that were not apparent at the behavioural level.

### 4.5 Conclusion

Our research strengthens evidence that recently abstinent individuals with AUD display negative alcohol‐related implicit cognition. In addition, leveraging preregistration and Bayesian analyses, we provide robust evidence that cathodal tDCS to the left PFC does not modulate implicit associations in this population. These findings significantly direct future research away from single‐session protocols and towards exploring the potential of multi‐session stimulation paradigm, which may induce the necessary neuroplastic changes. In contrast, the observed mitigation of nicotine craving may be attributed to the immediate, primary effects of cathodal tDCS, suggesting its potential for addressing ongoing craving in the short term. Our results further highlight the link between recent addiction treatment, explicit cognition and implicit associations at a critical phase of addiction recovery, indicating that implicit and explicit cognition undergo a dynamic, context‐dependent interaction. Future research might investigate the temporal dynamics of this interaction during the addiction cycle, which could help detect periods of higher vulnerability for relapses or identify optimal time points for targeted psychotherapeutic or neuromodulatory intervention.

## Author Contributions

P.A.S., T.S. and C.P. were responsible for the study concept and design. J.P. and T.S. contributed to the acquisition of behavioural data. P.A.S., T.S., S.W. and J.P. assisted with data analysis and interpretation of findings. T.S. and J.P. drafted the manuscript. P.A.S., S.W. and C.P. provided critical revision of the manuscript for important intellectual content. All authors critically reviewed content and approved the final version for publication.

## Disclosure

During the preparation of this work, T.S. utilized Microsoft Copilot to enhance the English language usage. No AI‐assisted technology was used for data analysis or for writing the result or method section of the manuscript. Following the use of this tool, the authors conducted a thorough review and made necessary edits to the content. The authors bear full responsibility for the content of the publication.

## Ethics Statement

The study was approved by the local Ethics Committee (877/2017BO2) at the University Hospital Tübingen, Germany.

## Consent

All participants signed written informed consent prior to study inclusion and any experimental procedures.

## Conflicts of Interest

The authors declare no conflicts of interest.

## Supporting information




**Figure S1.** Example of alcohol approach Implicit Association Test (IAT)
**Table S1.** Alcohol approach IAT & drinking identity IAT ‐ Semantic Stimuli
**Table S2.** Alcohol approach IAT ‐ pictorial stimuli
**Table S3.** Flower insect IAT ‐ pictorial stimuli
**Table S4.** Results for preregistered ANOVAs

## Data Availability

Data and analysis scripts supporting the results are openly available at OSF: https://osf.io/wd6em/files/osfstorage.
