# Supplementary material for: Cathodal Transcranial Direct Current Stimulation Does Not Change Implicit Associations Against Alcohol in Alcohol Use Disorder: A Preregistered Clinical Trial
Source: Addict Biol. 2025 Mar 12;30(3):e70029. doi: 10.1111/adb.70029 (PMC11899756; doi:10.1111/adb.70029)
Supplement: Supplementary file 1 — Figure S1. Example of alcohol approach Implicit Association Test (IAT) Table S1. Alcohol approach IAT & drinking identity IAT ‐ Semantic Stimuli Table S2. Alcohol approach IAT ‐ pictorial stimuli Table S3. Flower insect IAT ‐ pictorial stimuli Table S4. Results for preregistered ANOVAs [file ADB-30-e70029-s001.docx]

**Supplementary Material**

**Cathodal transcranial direct current stimulation does not change implicit associations against alcohol in alcohol use disorder: A preregistered clinical trial**

Tobias Schwippel^1,3,4*^, Philipp A. Schroeder^2*^, Janik Philipp^1^, Simone Weller^1,5^, Christian Plewnia^1,5^

*Authors contributed equally

1 Department of Psychiatry and Psychotherapy, Neurophysiology & Interventional Neuropsychiatry, University of Tübingen, Calwerstraße 14, 72076 Tübingen, Germany

2 Department of Psychology, University of Tübingen, 72076 Tübingen, Germany

3 Department of Psychiatry, University of North Carolina at Chapel Hill, Chapel Hill, NC 27599, USA

4 Carolina Center for Neurostimulation, University of North Carolina at Chapel Hill, Chapel Hill, NC 27599, USA

5 German Center for Mental Health (DZPG), Germany

**Supplementary Methods**

1. IAT Task

Figure S1. Example of alcohol approach Implicit Association Test (IAT)


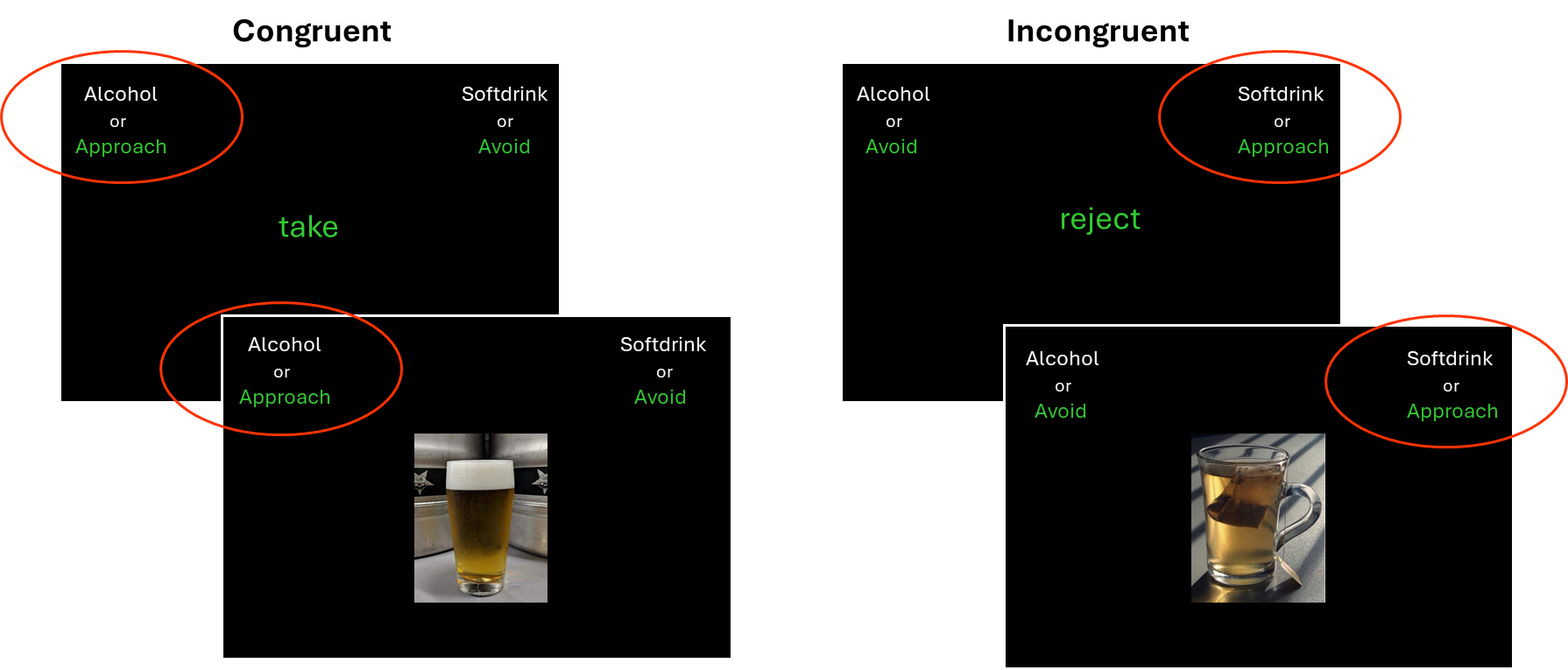


Figure S1: Example of an alcohol approach IAT. Encircled words are instructions visible throughout the entire task. In the screens center, test stimuli (pictures or words) are presented in a randomized order. The task was conducted in German language.

1. IAT Stimuli

For this study, semantic stimuli for the IATs were generated in German following two considerations. First, stimuli should be semantically close to previous studies. Second, we selected words with comparable count frequencies to account for salience asymmetries as a possible mechanism underlying the IAT and its modulation by cathodal tDCS (Rothermund & Wentura, 2004; Schroeder et al., 2017).

For the alcohol approach IAT we decided to use pictorial stimuli, thus differing from our previous experiment (Schwippel et al., 2022). This was based on findings from Foroni and Bel-Bahar who reported a stronger IAT response for pictorial compared to semantic stimuli (Foroni & Bel-Bahar, 2010). The pictorial stimuli were selected from the internet under a Creative Commons License. The semantic stimuli from the alcohol approach IAT associated with avoid or approach were the same used in the previous experiment (Schwippel et al., 2022).

For the drinking identity IAT, we translated stimuli from Lindgren and colleagues into German (Lindgren et al., 2013) and used the same semantic stimuli which were used in our previous study (Schwippel et al., 2022). For the flower insect IAT, pictorial stimuli were adapted from Greenwald and colleagues (Greenwald et al., 2003) and again did not differ from our previous experiment (<https://onlinelibrary.wiley.com/doi/10.1111/adb.13180>). All semantic and pictorial stimuli used in the IAT are listed in tables S1, S2 and S3.

Table S1: Alcohol approach IAT & drinking identity IAT - Semantic Stimuli

|  | Stimulus | # Syllable | # in German corpus | English translation |
| --- | --- | --- | --- | --- |
| Positive  Approach avoidance | Humor | 2 | 8041 | Humor |
|  | Liebe | 2 | 36648 | Love |
|  | Sommer | 2 | 106143 | Summer |
|  | Frieden | 2 | 26468 | Peace |
|  | Paradies | 3 | 2924 | Paradise |
| Negative  Approach avoidance | Verlust | 2 | 18625 | Loss |
|  | Gewalt | 2 | 41352 | Violence |
|  | Schmerz | 1 | 4773 | Pain |
|  | Angst | 1 | 47446 | Anxiety |
|  | Elend | 2 | 1714 | Misery |
| Positive  drinking identity | Enthaltsamer | 4 | - | abstinent person |
|  | enthalten | 3 | 18425 | abstinent |
|  | trocken | 2 | 13178 | dry |
|  | nüchtern | 2 | 1782 | sober |
|  | Abstinenzler | 4 | 8 | abstinent person |
| Negative drinking identity | berauscht  betrunken | 2  3 | 257  3604 | intoxicated  drunk |
|  | Partylöwe | 4 | 15 | Party animal |
|  | Alkoholiker | 5 | 403 | alcoholic |
|  | trinken | 2 | 8201 | drinking |
| Approach | berühren | 3 | 2076 | touch |
|  | nehmen | 2 | 96876 | take |
|  | greifen | 2 | 20028 | grab |
|  | anfassen | 3 | 760 | grasp |
|  | holen | 2 | 25237 | get |
|  | annähren | 3 | 627 | approach |
|  | wollen | 2 | 227748 | want |
|  | wünschen | 2 | 18088 | desire |
| Avoid | vermeiden | 3 | 23607 | avoid |
|  | wegschieben | 3 | 17 | push |
|  | entfernen | 3 | 8937 | remove |
|  | flüchten | 2 | 5825 | escape |
|  | ausweichen | 3 | 5448 | evade |
|  | verschwinden | 3 | 8396 | disappear |
|  | lassen | 2 | 220973 | allow |
|  | meiden | 2 | 3060 | evade |
| Me | mir | 1 | 154013 | me |
|  | mich | 1 | 194426 | myself |
|  | meins | 1 | 281 | mine |
|  | Ich | 1 | 442235 | I |
|  | selbst | 1 | 298982 | self |
| Not me | sie | 1 | 1641876 | they |
|  | deren | 2 | 95715 | their |
|  | seins | 1 | 133 | his |
|  | andere | 3 | 250735 | other |
|  | diese | 2 | 456726 | these |

Word frequency count retrieved from https://www.wortschatz.uni-leipzig.de/de (June 2024)

Table S2: Alcohol approach IAT - pictorial stimuli

| **Soft-Drink** | **Alcoholic** |
| --- | --- |
| 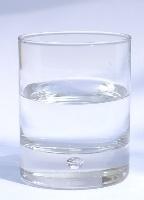 | 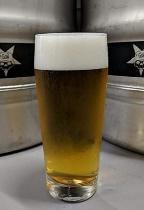 |
| 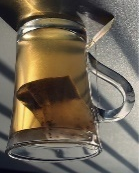 | 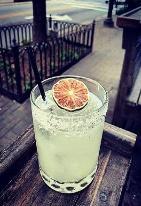 |
| 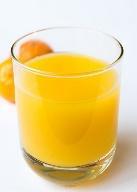 | 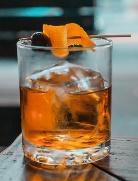 |
| 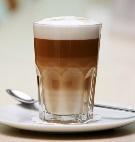 | 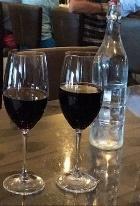 |
| 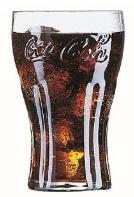 | 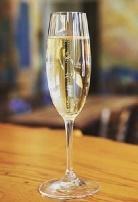 |
| 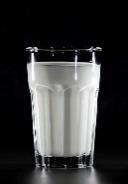 | 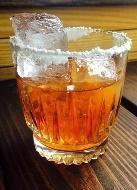 |
| 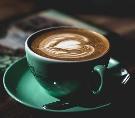 | 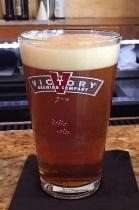 |
| 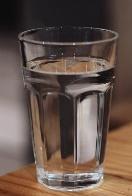 | 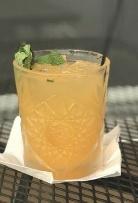 |

Table S3: Flower insect IAT - pictorial stimuli

| **Flower** | **Insect** |
| --- | --- |
| **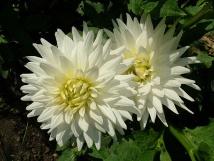** | **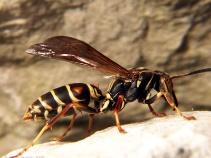** |
| **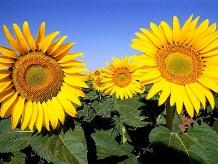** | **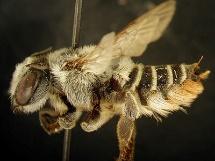** |
| **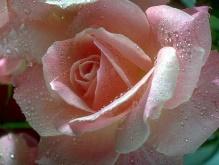** | **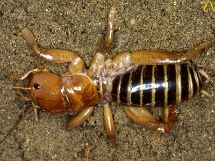** |
| **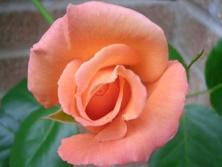** | **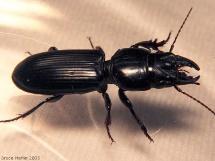** |
| **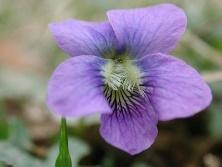** | **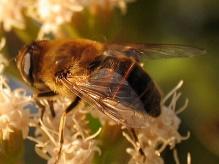** |

**Supplementary Results**

1. Preregistered ANOVAs – Investigating effects of stimulation condition

The following table displays the results of the preregistered ANOVAs, elucidating the main effects and interactions of IAT conditions and cathodal tDCS effects. All analyses were preregistered at OSF.
Link: [OSF Registries | Implicit measures of alcohol approach and drinking identity in alcohol use disorder: A preregistered double-blind randomized trial with 2mA cathodal transcranial direct current stimulation (tDCS)](https://osf.io/wd6em)

Table S4: Results for preregistered ANOVAs

| Effect | *F*-Value | *p*-Value | *η_p_²* |
| --- | --- | --- | --- |
| **Stroop Task** |  |  |  |
| Condition | (1,29) = .31 | .585 | .01 |
| Stroop | (1,29) = 98.93 | < .001 | .77 |
| Condition x Stroop | (1,29) = 2.81 | .105 | .09 |
| **Alcohol approach IAT** |  |  |  |
| tDCS | (1,28) = .00 | .998 | < .01 |
| IAT | (1,28) = 15.16 | .001 | .35 |
| Trial Type | (1,28) = 33.14 | < .001 | .54 |
| tDCS x IAT | (1,28) = .95 | .338 | .03 |
| tDCS x Trial Type | (1,28) = .01 | .920 | < .01 |
| IAT x Trial Type | (1,28) = .03 | .859 | < .01 |
| tDCS x IAT x Trial Type | (1,28) = .75 | .393 | .03 |
| **Drinking identity IAT** |  |  |  |
| tDCS | (1,29) = .05 | .833 | < .01 |
| IAT | (1,29) = 8.11 | .008 | .22 |
| Trial Type | (1,29) = 41.89 | < .001 | .59 |
| tDCS x IAT | (1,29) = .05 | .817 | < .01 |
| tDCS x Trial Type | (1,29) = 2.26 | .144 | .07 |
| IAT x Trial Type | (1,29) = 3.55 | .069 | .11 |
| tDCS x IAT x Trial Type | (1,29) = .18 | .677 | .01 |
| **Flower insect IAT** |  |  |  |
| tDCS | (1,29) = .097 | .333 | .03 |
| IAT | (1,29) = 66.26 | < .001 | .70 |
| Trial Type | (1,29) = 334.79 | < .001 | .92 |
| tDCS x IAT | (1,29) = .08 | .783 | < .01 |
| tDCS x Trial Type | (1,29) = .14 | .714 | < .01 |
| IAT x Trial Type | (1,29) = 1.22 | .278 | .04 |
| tDCS x IAT x Trial Type | (1,29) = 1.20 | .282 | .04 |

- 1. Preregistered D-IAT Score Analyses
     1. Alcohol approach IAT

In the analysis of D-IAT scores, a paired t-test was conducted to compare the effects of sham and cathodal tDCS, with D-IAT scores as the dependent variable and tDCS condition (sham vs. cathodal) as the independent variable. The results showed no significant difference between the two conditions, *t*(26) = -0.17, *p* = 0.866, *d* = -0.044.

- - 1. Drinking identity IAT

In the analysis of D-IAT scores, a paired t-test was conducted to compare the effects of sham and cathodal tDCS, with D-IAT scores as the dependent variable and tDCS condition (sham vs. cathodal) as the independent variable. The results showed no significant difference between the two tDCS conditions, *t*(26) = -0.77, *p* = 0.443, *d* = -0.124

- - 1. Flower-insect IAT

In the analysis of D-IAT scores, a paired t-test was conducted to compare the effects of sham and cathodal tDCS, with D-IAT scores as the dependent variable and tDCS condition (sham vs. cathodal) as the independent variable. The results showed no significant difference between the two conditions, *t*(26) = .221, *p* = .827, *d* = .039.

**Supplementary References**

Foroni, F., & Bel-Bahar, T. (2010). Picture-IAT versus Word-IAT: Level of stimulus representation influences on the IAT. *European Journal of Social Psychology*, *40*(2), 321–337. https://doi.org/10.1002/ejsp.626

Greenwald, A. G., Nosek, B. A., & Banaji, M. R. (2003). Understanding and using the Implicit Association Test: I. An improved scoring algorithm. *Journal of Personality and Social Psychology*, *85*, 197–216. https://doi.org/10.1037/0022-3514.85.2.197

Lindgren, K. P., Neighbors, C., Teachman, B. A., Wiers, R. W., Westgate, E., & Greenwald, A. G. (2013). I Drink Therefore I am: Validating Alcohol-related Implicit Association Tests. *Psychology of addictive behaviors : journal of the Society of Psychologists in Addictive Behaviors*, *27*(1), 1–13. https://doi.org/10.1037/a0027640

Rothermund, K., & Wentura, D. (2004). Underlying processes in the implicit association test: Dissociating salience from associations. *Journal of Experimental Psychology. General*, *133*(2), 139–165. https://doi.org/10.1037/0096-3445.133.2.139

Schroeder, P. A., Nuerk, H.-C., & Plewnia, C. (2017). Switching between Multiple Codes of SNARC-Like Associations: Two Conceptual Replication Attempts with Anodal tDCS in Sham-Controlled Cross-Over Design. *Frontiers in Neuroscience*, *11*, 654. https://doi.org/10.3389/fnins.2017.00654

Schwippel, T., Schroeder, P. A., Hasan, A., & Plewnia, C. (2022). Implicit measures of alcohol approach and drinking identity in alcohol use disorder: A preregistered double-blind randomized trial with cathodal transcranial direct current stimulation (tDCS). *Addiction Biology*, *27*(4), e13180. https://doi.org/10.1111/adb.13180
